# Annual Address before the Mississippi Valley Dental Society, by the President, Dr. F. H. Rehwinkle

**Published:** 1873-04

**Authors:** 


### Annual Address before the Mississippi Valley Dental Society, by the President, Dr. F. H. Rehwinkle.

*Gentlemen of the Mississippi Valley Dental Association:* Again have we assembled in this, to us all, familiar and dear old hall. We have come from the East and from the West, the North and the South, to enjoy once more the privilege of participating in this our annual reunion. Once more have we exchanged greetings with friends and brethren, and felt the pressure of the warm hands of many, whom we see but once each year. A year!—how long! when a space of time, composed of so many months, weeks and days, intervenes between us and the object of our wishes; and yet how short when past. It seems as if but a few weeks has elapsed since we said to each other in this very place, "Farewell! Farewell, until we meet again."

Through the blessing of our Heavenly Father, who vouchsafed us life and health, we have found our way again into this council chamber, where we are wont, not only to deliberate, but to work for the honor and welfare of our chosen profession. Let us all cheerfully do our alloted share.

We will in the first place take a slight retrospective view of the state of the dental profession. I believe it would be in vain to look for one among us, who is not willing to acknowledge that in many respects we are neither standing still nor retrograding, but are "marching on." None will fail to see that the operative department is steadily advancing to perfection. If we compare the general result of this branch, the character and qualities of operations of to-day, with those of only a few years since, we must admit that good and durable gold fillings are now the rule, instead of the exception; that many thousand teeth are now being succesfulsly preserved, which but a short time since were sacrificed to a supposed necessity; thus calling for artificial substitutes. The eye is daily gladdened by the sight of operations, which to the man of average ability, ten or even five years ago, were simply impossible.

Let us therefore render all due acknwledgements to those who have given us the neans of accomplishing results so satisfactory. What and where would we be without Barnum's "rubber dam?" Would those of you who are using mechanical power in dental operations, such as burring engines, electrical or automatic pluggers, together with numerous other late auxiliaries, willlingly be deprived of their use? Last, but not least, we have our faithful allies, the "gold beaters." Side by side have they marched with us in this progress toward perfection in the making gold fillings. There has been among them a constant spirit of emulation as to who could best develop the manifold qualities of the material in question, and present it to us in the most suitable and convenient form for use.

Dentistry in its scientific development is making fair and promising progress. Able and earnest men through the aid of the microscope, are investigating every tissue of the human body, and are adding certainty to points hitherto less clear, and to a great extent hypothetical. Physiologists and pathologists, not idle, are industriously collecting, comparing, and recording results. And when human knowledge seems to be in doubt or at a stand still, "Inspiration," we are told, "opens the eyes of its favorites;" thus making them the medium of expounding mysteries, correcting errors, and rending the veil which yet hides many things from the eyes of ordinary mortals. Through the aid of such instrumentalities it is but reasonable to hope that ere long our brethren across the water will have no occasion to say: "that although America may perhaps excel as far as practical dentists are concerned, in a scientific point of view, she must, for the present, take a back seat."

I have presented to your view some established facts, highly gratifying as regards the present, and with reference to the future, stimulating. Thus far all seems well with us; but while we have reason to congratulate ourselves upon achievements in one branch, let us see how stands the other, I allude to the mechanical department; I fear there is nothing encouraging to be said on this subject. To my view it presents itself as a bad omen, that by common consent, as it were, we have, and are considering it merely a question of time, as to when the actual separation of one branch from the other shall take place. Why is it that most of our best dentists have become disgusted with it, and as much as possible avoid the laboratory? Are not a great many of our brethren almost ready to abandon it altogether, were it not, that by so doing, they would virtually surrender this branch entirely to dental charlatans and huxters? These questions can be answered by every one present. The causes which have led to this state of things are so plainly to be seen around us, that I will not waste time by specifying each and all, preferring to call your attention to the principal facts which have arrested progress in this department, and to a consideration of how far we are all answerable for the same.

You recollect when hard rubber and kindred materials as bases for artificial dentures made their appearance, they were hailed by the profession as valuable *auxillaries only*. Who would have thought that in a few years the more difficult, but truly artistic work of platina, porcelain, and gold, would be looked upon by many as things of the past? Who would have imagined that blind enthusiasm would cause the master to think it "not worth while" to teach this kind of work any longer? Was it to be supposed that dentists would be found a few years later who were utterly incapable of constructing a metalic plate? Would you not have laughed to scorn the prophet who would have foretold that the introduction of these new materials would eventuatly have a demoralizing effect upon the whole profession? I said that all of us must take our share of blame; for did we not all, more or less, abandon the use of metalic bases for artificial plates, especially when the high premium on gold furnished us, for a time at least, an excuse for using *substitutes?* Artistic work and low prices are incompatible, and can not exist together. A quotation from Josiah Wedgwood, which I found in a pamphlet by James W. White, on "taking impressions of the mouth," published by S. S. White, "forcibly and admirably contrasts excellence and cheapness, and is so applicable to every variety of cheap dentistry," that I give the quotation in full:

"All works of taste must bear a price in proportion to the skill, taste, time, expense and risk attending their invention and manufacture. Those things called dear are, when justly estimated, the cheapest; they are attended with much less profit to the artist than those which everybody calls cheap. Beautiful forms and compositions are not made by chance, nor can they ever, in any material, be made at small expense. A competition for cheapness, and not for excellence of workmanship, is the most frequent and certain cause of the rapid decay and entire destruction of arts and manufacture."

Vain has been the earnest and loud protest of our best men against a continuance of this suicidal course. Vain has been the zealous endeavor to enlighten the public as to its own best interests. "Dental slaughter houses," as friend Osmond calls them, on the one hand, and "tooth carpenter shops," called steam dental establishments, on the other; the one to rob the mouth of teeth which might be saved with even ordinary skill, and the other supplying the loss with sets of "store-teeth," for from five to ten dollars, seem to have had the advantage. Consider too the numberless dental huxters, going from house to house, tearing out every tooth they can lay their hands on; here cooking up a set of teeth on rubber, and there on gun cotton and camphor.

Gentlemen, mechanical dentistry cries aloud for succor and support, and demands of us its preservation as an art. As such, it is worthy and deserving of our best efforts; looked upon as a trade, it needs no help—being well able to take care of itself. This subject will be presented for discussion in the regular order of business, and to it I invite your earnest attention.

It is equally desirable to secure to our profession a more dignified position in the estimation of our colleagues abroad. Our national associations, to which we might naturally look to conceive and mature measures, promotive of the interests of the whole dental profession in the United States, have thus far utterly failed to do, or suggest, anything conducive to this result; preferring to fritter their time away with affairs of personal or subordinate import. I think it therefore judicious and well-timed to suggest to this association, older than all others, the propriety of taking the initative in a matter of reform, demanded by justice, and called for on the part of its members, by true manhood and dignified modesty.

It is a matter of surprise and ridicule to English and other European dentists, that we in this country prefix the title of "Doctor" to our names, without having the slightest claim thereto. Our foreign brethren are often tempted to regard us as a set of presumptious pretenders, or vain and weak-minded fools. They will as a matter of course look upon our habits and customs from their own standpoint, and will fail to comprehend that titles are "lying around loose" in this country, and may be had for the picking up. We might tell them that not only dentists are in the habit of assuming or accepting this title, but almost every man who sells castor oil or tinct. of cinnamon, in due course of time, despite himself, gets a label with "Doctor" stuck on to him. Then those gentlemen, who visit us periodically with a "well selected stock of dental goods," submit with commendable resignation to their doctorate, conferred upon them by their house. The very urchins of our office, who wield the mallet, and strike the blows, are called "Doc" by their playmates in the street.

The American people are proverbial for their good nature and fun-loving proclivities, and frequently bestow titles in a humorous or frolicsome way, often intending them as "nick-names" only. If this could always be understood abroad, little harm would be done to professional dignity; and however much it may lessen the value of titles, it would explain the habit of bestowing them. It is not so easy to say why we, as professional men, should likewise have fallen into this careless way of giving the title of Doctor to every dentist without distinction; and here is where the seriousness of this thing forces itself upon our attention.

With diffidence I enter upon a further discussion of a subject so delicate in its nature, but trust that I may possess sufficient moral courage to speak plainly and fearlessly of a matter, which necessarily deeply influences our professional standing.

Whether this condition of things is a relic of the time when Dentistry was a mere handicraft, struggling into professional life; whether the title was *bestowed* by the people, or *assumed* by individuals, it is immaterial to know. Our chief concern should be as to how much we severally have aided in perpetuating a custom, no longer in accordance with the times, and detrimental to our true interests as members of a learned profession.

Let us take up the proceedings of dental societies in this country; there is no difference in either local, state, or national associations. Each person is addressed and reported as "Doctor." The title is bestowed indiscriminately, and accepted by all; there is no record of a single instance where a disclaimer to the right thereof was made by any one, to my knowledge. These facts go abroad, and bear witness against us.

I should not weary you, my colleagues, with a lengthy definition of the meaning of the word "Doctor;" in connection with my remarks, it is enough for us to remember that it is an honorary distinction—a degree—conferred by a duly authorized college, either of medicine or dental surgery. Of the latter institutions I think we have eight or nine in this country now; and by these the degree of D. D. S. is legally conferred. Now gentlemen, is it right or just that we should constantly help to render meaningless honors and distinctions, granted and bestowed by said colleges upon their graduates, as a legal recognition of meritorious qualifications? Is it just to the graduates from these schools practically to demonstrate to them, that their degree of D. D. S., and honors connected therewith, and for which they have toiled, and it may be, suffered many privations, amount to nothing?—merely a sham?—because the assumed title receives as ready recognition as theirs, not only from the public but from the members of their own profession!

Finally is it in accordance with true manhood and self-respect to appropriate honors and titles to which we have no claims?—setting up false pretenses, and occupying positions at once questionable?

No wonder that the profession in Europe does not understand this state of things, and looks upon it—to put it mildly—as a strange defect of our otherwise fair professional character.

Before me I have the "Deutsche Vierteljahresschrift fuer Zahuheilkwnde," organ of the Central Union of German Dentists. In looking over the proceedings of this Association, the nice discrimination and scrupulous exactitude of giving every one his due is most striking. Here we find the names of men who are shining lights in their profession, and of world wide reputation, recorded as: Herr Ed. Muehlreiter, Fahnarzt, in Salzburg, (this gentleman is the present editor of this journal, and much esteemed as a learned and scientific man). Then we find Herr Adolph zur Nedden, Zahnarzt, in Nuernberg, well known as the former editor of this journal; the translator of "Taft's Operative" and "Richardson's Mechanical Dentistry;" a man of great worth, and of high repute, now dead. We have also men of titles: Herr Hofrath, Doctor Wm. Sueerson, Zahnarzt, Berlin; Herr Prof., and Dr. von Weltz, Kt., etc., Zahnarty, Wuerzburg; Herr Dr. von Langsdorff, Zahnarzt, Freiburg; this gentlemen is a pupil of our own Atkinson, and spent several years in this country. He advocates the advantages of our system of dental education; defends us when assailed, and explains and apologises for our "eccentricities."

In this way I might go on and read to you the list of active members, some hundred and twenty. In recording the discussions you will see, Herr Schmidt, or colleague Brown, or Herr Dr. Augenzahn, said so and so.

What English dentists think about this loose way of ours, you may learn from that renowned surgeon dentist, Charles James Fox, Esq. I quote his own words, taken from the "*Dental Cosmos*, for June, 1872, page 325.

No. 27 Mortimer St., Cavendish Square,
London, May 3d, 1872.

*To the Editor of the Dental Cosmos:*

"Dear Sir:—Will you be good enough to remove the *Sir* which has somehow been attached to my name after a short notice of Guillois' cement, which has appeared in your advertisements the last month or two? I did not consider it worth while writing about at first, deeming it a printer's error, which would not be repeated, but as it *has* been repeated more than once, I must beg you to allow me to disclaim in your pages, having any right to the title, and again beg you to remove it. As it is customary with many Americans to address all dentists as Doctor, it may possibly be a misprint for that; but as I hold the English diplomas of M. R. C. S. and L. D. S., and not the American degree of D. D. S., I am not entitled even to the title of Doctor."

I am Dear Sir, yours faithfully,

Charles James Fox.

Comments upon the letter just quoted, would only lessen its force; it is complete and speaks for itself. Would it not be well for us all to look upon it as an example most worthy of imitation?

This Association, with its most liberal policy towards its members, seems to me above all others, best fitted to inaugurate a movement of reform in its own midst, without wounding the feelings of any one. We all know that this venerable old society, the mother of the Ohio College of Dental Surgery, has always thrown its doors wide open to every comer; giving everything, exacting nothing. An honorable character as a dental practitioner was the only thing requisite to gain admission, and enjoy full membership. Men of learning become the voluntary and gratuitous teachers of those less favored. It has always been a nursery for the young and feeble plant; giving it shelter and support. No one cared whether it was plain Mr. Scheider, Dentist, or Herr Hofrath, Dr. Weisheitszohn, who applied for membership. Thus may it ever be; may the M. V. A ever be in the "vanguard" of liberality to the members of the dental profession; but may it also be just and true to its own dignity.

In presenting the foregoing remarks to you, let me hope, gentlemen, that I have discharged a duty without wounding your pride; I beg to assure you, that only the most anxious solicitude for the welfare and honor of the dental profession at large, had led me to choose a subject, in itself not popular. I have not allowed my mind to become prejudiced by individual cases, however exasperating, and all material which they might have furnished has been scrupulously rejected. I stand before you then, with the best motives, and if I appeal to your sense of justice to lend a helping hand in this matter, let me not plead in vain.

